# Therapeutic bacteria and viruses to combat cancer: double-edged sword in cancer therapy: new insights for future

**DOI:** 10.1186/s12964-024-01622-w

**Published:** 2024-04-24

**Authors:** Aref Yarahmadi, Mitra Zare, Masoomeh Aghayari, Hamed Afkhami, Gholam Ali Jafari

**Affiliations:** 1 Department of Biology, Khorramabad Branch, Islamic Azad University, Khorramabad, Iran; 2 Department of Microbiology, Faculty of Sciences, Kerman Branch, Islamic Azad University, Kerman, Iran; 3 Department of Microbiology, Faculty of Sciences, Urmia Branch, Islamic Azad University, Urmia, Iran; 4 Nervous System Stem Cells Research Center, Semnan University of Medical Sciences, Semnan, Iran; 5 Cellular and Molecular Research Center, Qom University of Medical Sciences, Qom, Iran; 6 Department of Medical Microbiology, Faculty of Medicine, Shahed University, Tehran, Iran

**Keywords:** Cancer, Bacteria, Viruses, Immunotherapy, Therapy

## Abstract

Cancer, ranked as the second leading cause of mortality worldwide, leads to the death of approximately seven million people annually, establishing itself as one of the most significant health challenges globally. The discovery and identification of new anti-cancer drugs that kill or inactivate cancer cells without harming normal and healthy cells and reduce adverse effects on the immune system is a potential challenge in medicine and a fundamental goal in Many studies. Therapeutic bacteria and viruses have become a dual-faceted instrument in cancer therapy. They provide a promising avenue for cancer treatment, but at the same time, they also create significant obstacles and complications that contribute to cancer growth and development. This review article explores the role of bacteria and viruses in cancer treatment, examining their potential benefits and drawbacks. By amalgamating established knowledge and perspectives, this review offers an in-depth examination of the present research landscape within this domain and identifies avenues for future investigation.

**Graphical Abstract:**

The double-edged sword role of bacteria and viruses in cancer therapy.

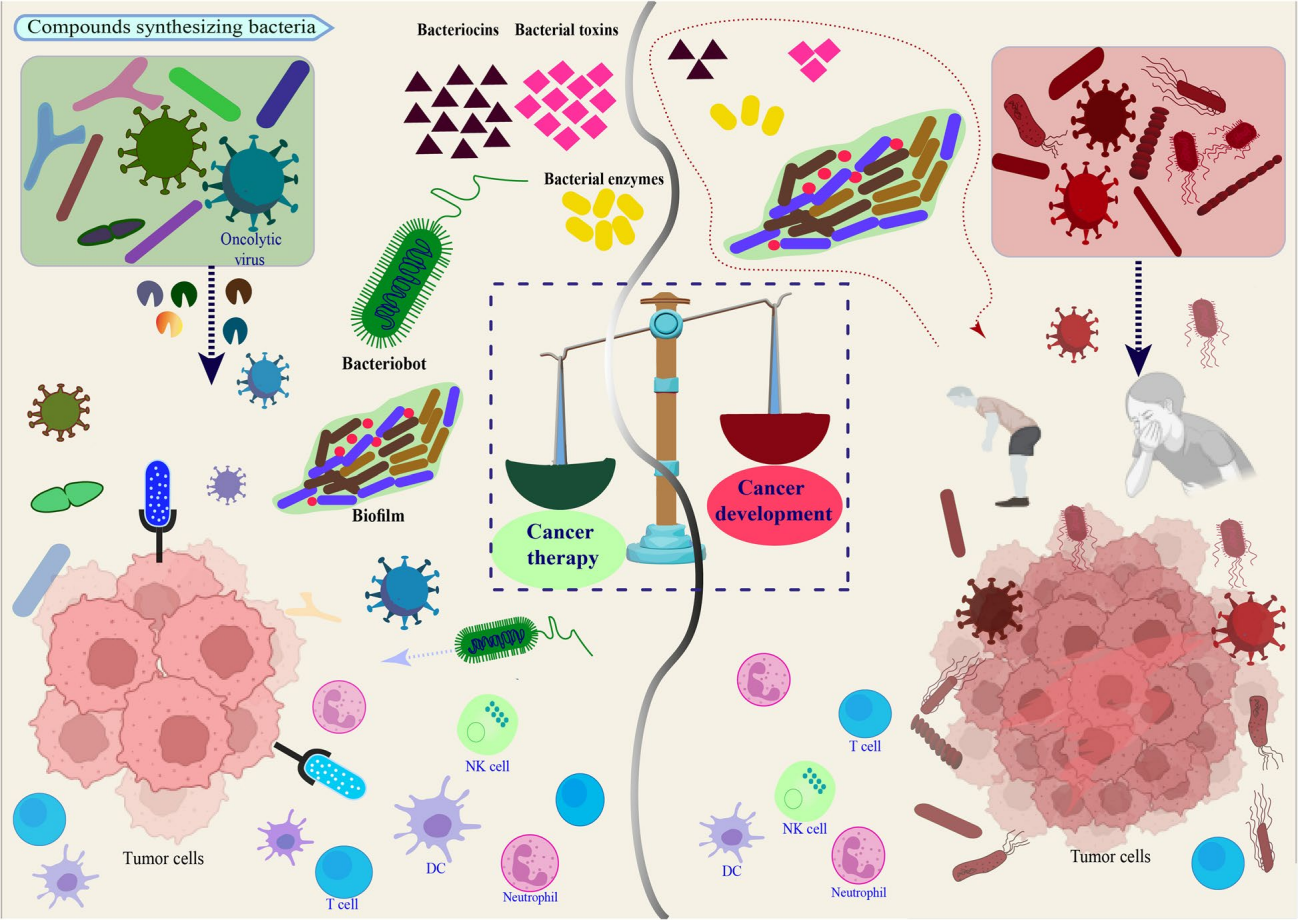

## Introduction

Cancer, being the second leading factor of mortality globally, claims the lives of approximately seven million individuals annually, thereby establishing itself as one of the most pernicious ailments worldwide [1, 2]. The unearthing and recognizing novel anti-cancer medications that eradicate or render cancerous cells inactive without inducing harm in sound and typical cells have reduced adverse impacts on the immune system. It has a potential challenge in medicine and medicine and is an essential goal in many studies. Cancer is a disease in which a cell or group of cells exhibits uncontrolled growth (i.e., division beyond normal), invasion (i.e., invasion and distortion of adjacent tissue), and metastasis (i.e., from one part of the body to another through the lymph or blood) these three characteristics distinguish cancer from benign tumors [2]. The survival rates for cancer have significantly risen since the beginning of the twenty-first century due to the development of more precise and enhanced treatment approaches. Approximately 609,820 individuals in the United States are projected to succumb to cancer in the year 2023, equating to an average of 1670 deaths per day. The highest mortality rates are attributed to prostate, lung, and colorectal cancers (CRC) in men and lung, breast, and CRC in women [1, 3]. The induction of apoptosis and the inhibition of tumor cell growth and proliferation have been the primary approaches employed in cancer therapy up until this point [4]. Alternative approaches to cancer treatment, including radiotherapy, chemotherapy, surgical intervention, and tumor extraction, have also proven to be beneficial in the management and recovery of patients. Nevertheless, these methods have shown limited efficacy in nearly 50% of cancer instances [5]. Chemotherapy medications have severe adverse effects and are toxic to all organs. Chemotherapy drugs are associated with severe side effects such as hair loss, bleeding, fatigue, sterility, cognitive defect, sensory anomalies, lung damage, Nervous Texture damage, liver damage, digestive system damage, etc. Emerging cancer medicine resistance is another serious problem with chemotherapy [6–8]. For numerous years, the quest for novel antitumor compounds with reduced side effects has been a matter of great importance. In this context, natural products derived from plants, marine Organisms, and microorganisms have attracted the attention of many scientists [9, 10]. Oncolytic virotherapy, an innovative technique in the field of cancer treatment, has exhibited encouraging outcomes over the past twenty years [11]. It was noted over one hundred years ago that individuals with cancer experienced a reversal of their disease when they contracted specific viral infections [12]. Most oncolytic viruses selected to treat cancer are either weakened strains or strains that can invade and reproduce within the human body without posing any significant risk of illness [13]. The investigation into the potential of bacteria as a viable method to combat and address cancer is a notable avenue being pursued within the field of immunotherapies [2, 14]. *Streptococci* and *Clostridia* were the initial types of bacteria employed as live agents in the fight against cancer. Currently, genetically engineered bacteria are predominantly used in anti-cancer therapies, wherein diverse anti-cancer mechanisms and strategies are utilized. These encompass live bacterial toxicity, the expression of distinct cancer-related factors, gene transfer, and RNA interference [2]. Tumor-targeting bacteria, such as *Salmonella*, *Listeria*, and *Clostridium* spp., have intrinsic properties that can target, penetrate, replicate, and shrink solid tumors through various mechanisms [15]. Once inside the tumor, *S. typhimurium* continues to multiply and directly kills and destroys cancer cells by inducing apoptosis, necrosis, and cell rupture [16–18]. Table 1 lists the cancers caused by Microorganism infections.

**Table 1:** Bacteria and viruses are associated with various forms of cancer

Microorganism		Cancer Type Associated	References
Viruses	Kaposi sarcoma-associated herpesvirus (KSHV)	Kaposi's Sarcoma (KS)	[19, 20]
	Human papillomavirus (HPV)	Esophageal adenocarcinoma [21], cervical cancer, oropharyngeal Cancer, gastrointestinal cancer (GI)	[22–26]
	Epstein Barr virus (EBV)	EAC, gastric cancer (GC), breast Cancer (BC), lung cancer (LC)	[22, 27–30]
	Merkel Cell Polyomavirus (MCPyV)	Merkel cell carcinoma (MCC)	[31, 32]
	Hepatitis B virus (HBV)	Liver cancer	[33]
	Herpes simplex virus (HSV)	EAC, cervical cancer	[22, 34]
	Cytomegalovirus (CMV)	BC, GI	[35–37]
	Hepatitis C virus (HCV)	Liver cancer	[38, 39]
	*Chlamydia psittaci*	Ocular lymphomas	[40, 41]
	*Mycobacterium tuberculosis*	LC	[42]
	*Helicobacter pylori* (*H. pylori)*	GC	[43–45]
	*Chlamydia pneumonia*	LC, lymphomas	[41]
	*Fusobacterium nucleatum (F. nucleatum)*	Colorectal cancer (CRC)	[46–48]
	*Bacteroides fragilis*	CRC	[49, 50]
Bacteria	*Escherichia coli (E. coli)*	CRC	[49, 51, 52]
	*Streptococcus bovis*	CRC	[53, 54]
	*Salmonella enterica serovar Typhi*	Gallbladder cancer	[55–57]
	*Campylobacter jejuni*	CRC, small intestinal lymphomas	[19, 20, 40, 58]
	*Chlamydia trachomatis*	Cervical cancer, epithelial ovarian cancer (EOC)	[41, 59, 60]
	*Porphyromonas gingivalis* (*P. gingivalis*)	GI, CRC, pancreatic cancer, oral cancer	[61–63]

*Abbreviations*: *GC* Gastric cancer, *BC* Breast cancer, *LC* Lung cancer, *CRC* Colorectal cancer

The study aims to investigate the role of bacteria and viruses in cancer treatment and the development and progression of cancer and to gain new insights for the future.

### Bacteria and viruses in cancer therapy

Bacteria and viruses have emerged as novel therapeutic entities in the battle against cancer. Utilizing these living entities as curative agents has a lengthy historical background [64–66]. These biological agents can directly assail and remove malignant cells or serve as a strategy to enhance the efficacy of additional pharmaceuticals in cancer therapy [67, 68]. Oncolytic bacteria and viruses, such as *Bifidobacteria*, *Clostridium*, *Listeria monocytogenes*, *Salmonella typhimurium*, *Bacillus*,* Vaccinia viruses*, *Adenoviruses*, *Reoviruses*, *Herpesviruses*, and *Coxsackieviruses*, have arisen as remarkable therapeutic strategies in the quest for the treatment and potential eradication of malignant tumors [69]. Due to their inherent anti-cancer properties and ability to interact with tumor microenvironments (TME), these microbes are attractive options for cancer therapy [70, 71]. Living viruses were administered to cancer patients in the 1950s and 1970s, which improved their course of treatment or recovery [72, 73]. For almost a century, several organizations have promoted using microorganisms to cure cancer [74]. William Coley pioneered in the early 1900s when it came to using microorganisms to treat cancer [12]. Additionally, it has been shown that some bacteria and viruses have evolved defense systems that obstruct cellular pathways and lessen hosts' capacity to heal damage, resulting in cellular transformation and the advancement of cancer [75–77].

### Virotherapy for cancer (oncolytic viruses and oncolytic viral vectors)

**Fig. 1:**
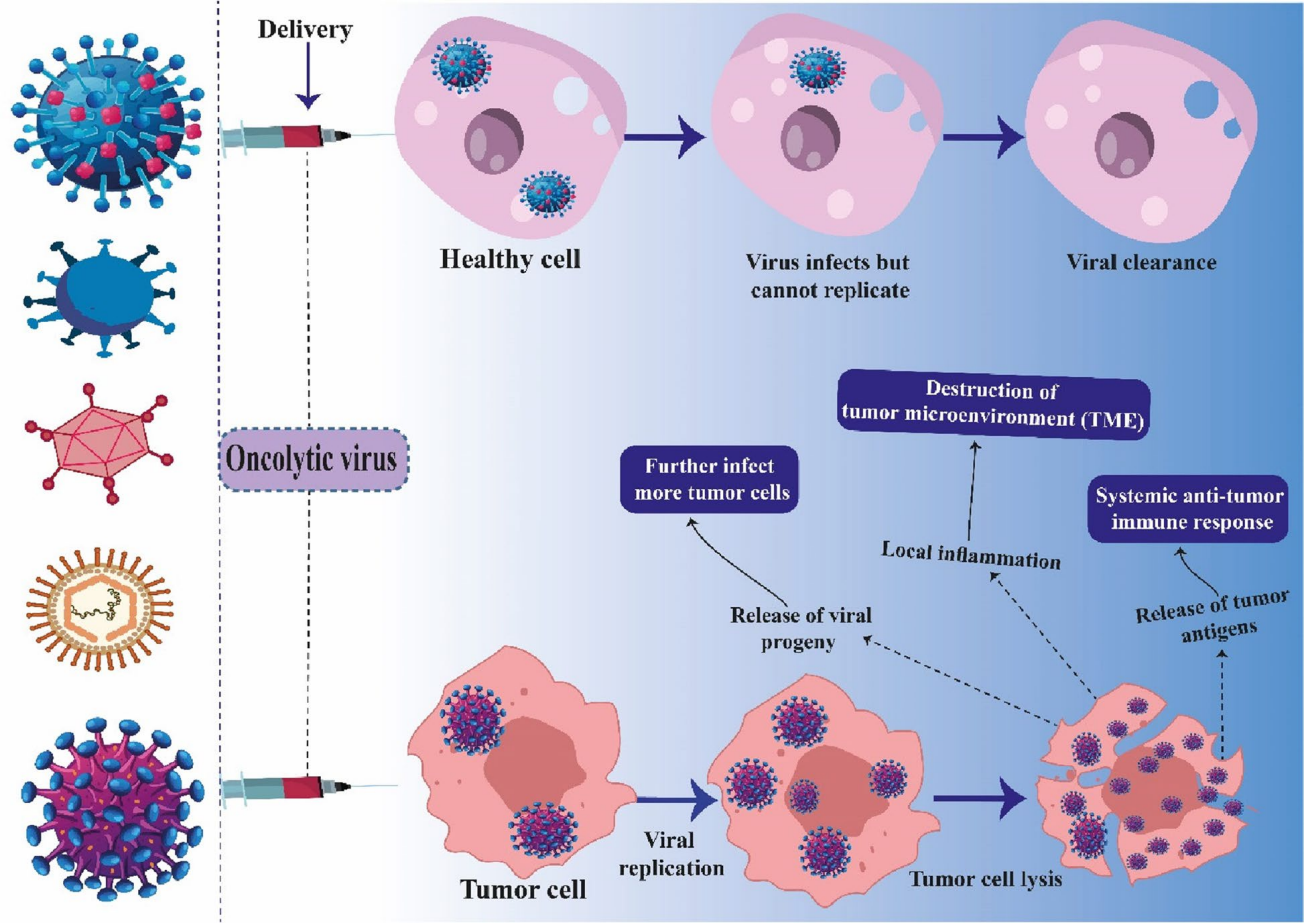
Virotherapy for cancer

Virotherapy is defined as the use of viruses in the diagnosis and treatment of chronic diseases, including infectious and noninfectious diseases such as central nervous system (CNS) disorders, genetic diseases, and cancers [78–80]. These viruses, called oncolytic viruses (OVs), are in different research levels, from basic molecular studies to clinical trials, and some of these efforts led to approved drugs [81]. These viruses can target and lyse malignant cells and enhance tumor regression (Fig. 1) [82]. The OVs can be classified into at least two categories: wild-type viruses and oncolytic viral vectors [83]. OVs exert their effects by targeting malignant cells and inducing their demise. One prominent illustration in this particular domain is the agent "talimogene laherparepvec," which has exhibited favorable outcomes when managing melanoma [84, 85]. The immunosuppressive TME presents a formidable obstacle to virotherapy [86]. The presence of low oxygen levels in the TME can have contrasting effects on the replication of OVs, either enhancing or inhibiting their proliferation, depending on the specific type of virus being considered [87]. Genetic manipulation or molecular alterations aimed at decreasing hypoxia possess the potential to augment antitumor reactions. The integration of OVs that can induce tumor lysis within the hypoxic TME may present a compelling approach for surmounting the constraints encountered in therapy [88]. For almost a century, the idea of OVs has been discussed. It was reported in 1904 that a 42-year-old woman's leukemic condition improved after contracting influenza. Later, in 1912, Italian doctors found that rabies vaccination injections might cause cervical cancer [81, 89, 90]. OVs have been used to treat multiple myeloma (MM), an incurable hematological disease. Human viruses have been studied; however, pre-existing anti-virus immunity limits their efficacy. Bovine viruses have demonstrated the ability to destroy MM cells directly, including Bovine Viral Diarrhea Virus (BVDV) and Bovine Herpes Virus type 1 (BoHV-1) [83, 91–94]. Tumor cells are selectively attracted to OVs, which promotes oncolysis and increased replication [95]. To get over treatment roadblocks, combinatorial therapy which involves trans genes like GM-CSF expressed in T-VEC has been investigated in addition to conventional therapies [96]. Numerous OVs have been studied for their methods of action and impacts on immunogenic cell death, apoptosis, autophagy, and immune system modulation. These include oncolytic vaccinia virus (OVV), vesicular stomatitis virus (VSV), and herpes simplex virus (HSV) [97]. The therapeutic potential of self-replicating RNA viruses, including flaviviruses, alphaviruses, rhabdoviruses, measles viruses, and others, has also been studied in cancer treatment [98].

### Bacteriotherapy for cancer

The potential of bacteria and their metabolites to destroy tumor cells as a novel anti-neoplastic technique has been investigated by scientists and researchers throughout the last ten years [14, 99]. They have discovered that on average, healthy cells, bacteria, and their products are not only less harmful but also have fewer adverse consequences [100, 101]. Patients with cancer have been treated with various bacterial species and their metabolites (peptides, bacteriocins, etc.) [102, 103]. The outcomes show that these substances can affect and selectively proliferate tumors while limiting their growth [104]. Almost all bacterial species emit cationic peptides called bacteriocins, produced in the ribosome [105, 106]. Certain bacteriocins exhibit a higher degree of toxicity towards cancerous cells than normal cells [107, 108]. Probiotic-derived probiotic-based medicines have demonstrated the potential to destroy cancer cells while sparing healthy cells from harm selectively [109, 110]. Certain bacteria can initiate infections within tumor tissue through biofilm formation. The infection triggers an immune response characterized by a swift influx of neutrophils to the site of infection [111–113]. Anaerobic bacteria spores possess the potential to be employed in the process of synthesizing, cultivating, and generating agents that exhibit anti-cancer properties. Gene and drug delivery to tumor tissues can also be facilitated by carriers [114–117]. These spores of bacteria can acquire hypoxic-necrotic tissues, in which they can sprout, reproduce, and demonstrate their antitumor role [118, 119].

### Through enhancing immunity (activating inflammasome pathways- CD4/8):

Bacterial constituents can amplify the interplay between tumors and the immune system, functioning as adjuvants. Adjuvants serve to invigorate the antigen and trigger the innate immune system [120]. Cancer immunotherapy encompasses activating a precise immune response within the patient, thereby enabling various categories of indigenous immune cells to target and combat cancer cells [121, 122]. The immune system consists of CD4 + and CD8 + T lymphocytes, which are activated after antigen stimulation by antigen-presenting cells (APCs) following the production of specific antibodies against the antigen [123]. CD8 + T-lymphocytes, macrophages, NK cells, dendritic cells (DCs), and regulatory T cells (T-regs) are the most pivotal constituents of the immune system that exert a profound influence on the suppression of malignant and aberrant cells. These immune cells possess FOXP3 as a biomarker, emphasizing their significance [124, 125]. CD8 + T-lymphocytes have gained recognition as the most prominent constituents of the immune system in their ability to impede the proliferation of cancerous cells [126, 127]. Each person has a unique immune system component with a different capability to mount an immune response [128]. Tumor-associated antigens on the surface of cancer cells occur with rapid cell proliferation and escape from the TME [129, 130]. Once the host's immune cells are activated (primarily tumor antigen-specific CD8 + and CD4 + T lymphocytes that are activated and stimulated), they can recognize and destroy tumor cells [131]. DCs, antigen-presenting cells, are required to generate effective immune responses [132]. Pathogen-associated molecular patterns (PAMPs) upregulate proinflammatory cytokines (such as IL-12) and inflammatory molecules (such as CD40) [133]. These *S. typhimurium flagellin* enhances the antitumor response of CD8 + T and natural killer (NK) cells. It decreases the frequency of regulatory T cells (Tregs( mediators induce the production of interferon-gamma (IFN-γ) and initiate a Th1-dependent cellular response that is primarily mediated by CD8 + effector cells [131, 134]. Tumor tissues may be suppressed by certain microbial infections and their consequences, such as infections brought on by *E. coli* [135]. These infections enhance and quicken CD8 + killer T-cell development, leading to the production of IFN-γ and an uptick in the expression of primary histocompatibility complex subtype I (MHC-I) on malignant cells. Finally, by using this integrative process, CD8 + T-cell diapedesis into tumor tissue may be altered [136]. It should be mentioned that CD8 + T cells can target malignant tissues even in the absence of bacterial infections or activities since their function in fighting tumor cells is known to occur independently of bacterial activity [137, 138]. Moreover, other substances linked to microbes may potentially impact CD8 + T cells. As an illustration, research by Diwakar Davar and colleagues [139] revealed that responder-derived fecal microbiota transplant (R-FMT) combined with pembrolizumab could enhance the induction of CD8 + T cells, decrease IL-8 synthesis, and bolster the immune responses against tumor cells resistant to anti-PD-1 [139, 140].

### The innate immune system ((TNF-α)) in bacteria

Tumor necrosis factor α (TNF-α) is a protumor factor in a chronic inflammatory environment, including tumors and many cancers [141, 142]. Phagocytic cells, including neutrophils, DCs, and macrophages, secrete TNF-α and are essential for limiting bacterial growth and triggering the production of more immune cells [143]. Through the NF-κB and AP-1 signaling pathways, TNF-α can cause inflammation by upregulating gene transcription [144, 145]. Additionally, involved in the pathophysiology of pulmonary illnesses, TNF-α plays a crucial role in host defense against intracellular pathogens such as *Mycobacterium tuberculosis* [146, 147]. TNF-α is also connected to neuroinflammation and the etiology of neurological disorders, such as infections and neurodegenerative illnesses [148, 149]. The multipurpose cytokine TNF-α is essential for controlling inflammation, cell death, and cell division [150, 151]. TNF-α is necessary for the development and spread of cancer. Research has revealed a strong correlation between TNF-α and lymphatic metastasis in cervical cancer. TNF-α activates vascular endothelial growth factor (VEGFC) mediated ERK and AKT pathways, promoting carcinogenesis, lymphangiogenesis, and lymphatic metastasis [141]. TNF-α activation improves the mesenchymality of breast cancer stem cells (BCSCs) in triple-negative breast cancer (TNBC), boosting their capacity for invasion, self-renewal, proliferation, and inducing intra-tumoral stromal invasion [152, 153]. Additionally, TNF-α stimulates stromal cells to produce matrix metalloprotease (MMP)-2, vascular endothelial growth factor (VEGF)-A, and colony-stimulating factor (CSF)-1, which promotes colon cancer carcinogenesis [154, 155]. Another treatment strategy to reduce angiogenic responses in the TME and stop secondary organ metastasis might be to target TNF-α [156, 157]. Knowledge of how TNF-α affects cancer growth and creating individualized treatment plans requires an understanding of how it interacts with other elements in the TME [158, 159]. The utilization of conventional TNF-α antibodies, which counteract the activity of TNF-α, produces a moderate antitumor outcome. In a syngeneic mouse melanoma experiment, the bacteria induced the upregulation of TNF-α, resulting in a synergistic effect with the secreted immunotoxin and significantly inhibiting tumor growth [160, 161]. This particular form of therapy restructured the TME in a manner that supported the presence of numerous immune cells with antitumor properties, such as M1 macrophages, N1 neutrophils, and activated CD4 + and CD8 + lymphocytes [162, 163].

### Combination of bacteriotherapy and immunotherapy

One therapeutic strategy for cancer called immunotherapy is predicated on strengthening the host immune system against malignancy [164, 165]. A variety of techniques are employed to block immune cells, such as immune checkpoint inhibitors (ICIs), adoptive cell treatments (such as CAR-T cells), monoclonal antibodies targeting tumor antigens, and the injection of cytokines [166, 167]. Specific techniques, such as the use of chemokine receptor inhibitors (CXCR4 antagonist AMD3100) and monoclonal antibodies (anti-CCR4 mAb, Mogamulizumab), are already being employed in clinical practice for hematological malignancies [168–170]. Immunotherapy modifies the expression of chemokine receptors in cancers, which controls the angiogenesis, proliferation, and recruitment of leukocytes into the tumor [171]. Targeting cytotoxic T lymphocyte-associated molecule-4 (CTLA-4), programmed cell death receptor-1 (PD-1), and programmed cell death ligand-1 (PD-L1), immune checkpoints are recognized as a significant and successful kind of immunotherapy [172]. Additionally, the primary goal of the immunotherapy strategy is to use Toll-like receptor (TLR) agonists, which are related to innate immune activation, to target the tumor's microenvironment [173, 174].

In anti-cancer bacteria-based immunotherapy techniques, the utilized bacteria may exist in a state of being alive or weakened and potentially even manifest as genetically modified variants [99, 175]. A cancer therapy mediated by a safe bacterium should possess characteristics like cytotoxicity towards cancer cells or immunogenicity, thereby minimizing harm to healthy cells, exhibiting a preference for cancer cells, and maintaining stability within the physiological conditions of the human body [176, 177]. Dr. William B. Coley (1936–1862), a bone sarcoma surgeon, pioneered the treatment of his patients with both live bacteria and a mixture of heat-killed bacteria known as " Coley’s toxins" [178, 179]. Following his remarkable discovery, many investigations have demonstrated remarkable outcomes when employing diverse bacterial strains to eliminate distinct types of tumors [180]. Despite the incredible outcomes achieved, the evolution of alternative therapeutic strategies, including radiation therapy and chemotherapy, led to the gradual obsolescence of Coley's toxins. Recent immunological research indicates that the overarching principles of Coley's toxins hold validity, as certain types of cancer exhibit an increased susceptibility to bolstering and optimizing the patient's immune system [181, 182]. Numerous bacterial species have demonstrated a fantastic capacity to penetrate and colonize solid tumors, a phenomenon that frequently results in the growth retardation of neoplasms and the removal of tumors [183, 184]. The genera *Bifidobacterium*, *Clostridium*, *Lactococcus*, *Shigella*, *Vibrio*, *Listeria*, *Escherichia*, and *Salmonella* were used in animal cancer models [185]. Obligate anaerobes such as *Bifidobacterium longumo* (*Clostridium novyi* non-lethal toxin strain) have been shown to kill tumors in mice after systemic administration in hypoxic necrotic areas, which in some cases causes tumor regression [186]. Although the growth of viable tumor tissue was impeded by the presence of high oxygen tension, the anti-cancer properties of an attenuated facultative anaerobic auxotrophic mutant of *Salmonella enterica serovar Typhimurium* arise from the biological interactions between the bacteria and the host tumor, both directly and through immune-mediated mechanisms [187, 188]. Bacteria-mediated tumor treatment (BMTT) has been used for a long time to manage cancer despite its adverse effects. When using BMTT, it's important to balance its therapeutic benefits against potential adverse effects, including infection [189–191]. The only bacterial agent the FDA has licensed to treat non-muscle invasive bladder cancer (NMIBC) since the late 1970s is Bacillus Calmette-Guerin (BCG), an attenuated strain of *Mycobacterium bovis*. For high-risk NMIBC, BCG has been the accepted standard of care and is the most successful therapy [192–194].

Bacteria as anti-cancer agents through amplification Human immunity interacts with the host as one of the pathogenic factors or natural flora strengthens the host's immune system in interaction with pathogenic bacteria [195]. The *Salmonella typhimurium* strain ΔppGpp impedes the signals that are sent, which initiates inflammatory pathways [196]. The amount of inflammatory cytokine IL-1β, TNF-α, and Il- 18 in tumors leads to severe tumor growth suppression; IL-18 plays an essential role in immunity against pathogens [184]. Anaerobic bacteria such as *Escherichia coli* (*E. coli*) can are involved in solid tumors, indirectly Clearance of some tumor cells through the CT26) infectious defense mechanism; when these bacteria invade their host, they trigger the initiation of defense. This mechanism leads to the production of host T lymphocytes involved in antitumor activity [136, 197]. T cells are the only agents responsible for tumor clearance [136, 184]. Recombinant *E. coli* K12-producing TNF-α, an anti-cancer cytokine that may directly kill cancer cells and trigger antitumor immunity, was created by Murphy et al. [198–202]. Research revealed that the genetically modified *E. Coli* K12 gathered within tumors in a specific manner and significantly decreased tumor loads [203]. In a different investigation, Lee et al. [199] assessed the capacity of recombinant *Salmonella* with an endostatin expression vector to target tumors and deliver genes. Angiogenesis inhibitor endostatin can prevent vascularization and limit tumor development [204]. The recombinant Salmonella was found to produce tumor regression, decrease tumor microvessel density, and colonize tumors preferentially [17, 205] (Table 2).

**Table 2:** Bacteriotherapy in the treatment of cancers

Type of Study	Country	Bacteria type	Sample	Cancer type	Conclusion	Reference
Cell culture	Iran	*Bifidobacteria*	colon adenocarcinoma cell line LS174T	CRC	It increases intestine length by stopping the tumor from growing to more prominent stages and worsening	Parisa et al. [206]
	USA	*Salmonella typhimurium* A1-R	Stomach adenocarcinoma cell line, MKN45	Stomach adenocarcinoma	Cancer cell corruption caused by* S. typhimurium* A1-R	Hoffman et al. [207]
	USA	*Listeria monocytogenes*	CT26 colon cancer cell line	Colon cancer	The creation of this vaccination platform in patients with liver metastases was made possible by the very efficient antitumor T-cell response elicited by this strain of *L. monocytogenes*	Olino et al. [208]
	China	*Bacillus subtilis*	Colon cancer HT29 cell line	Colon cancer	According to a study, fengycin targets the Bax/Bcl-2 pathway, which may have an inhibitory effect on cell apoptosis and cell cycle processes, ultimately contributing to the formation and spread of colon cancer	Cheng et al. [209]
	Australia	*Clostridium perfringens*	(DU145, PC3), (MCF7), (HUVEC), (A549), (SW480)	Skin epidermoid cancer cell line, LC cell line, Cervical cancer cell line, Human umbilical vein endothelial, Human prostate cancer cell, BC	The hybrid toxin effectively targets CLDN-4 positive cancer cells by binding to its receptor and causing apoptosis, suggesting potential for molecularly targeted treatment	Yao et al. [210]
		*Clostridium novyi*-NT	––––-	High gliomas -grade	These findings demonstrate the exact eradication of cancerous tissues by *Clostridium novyi*-NT	Roberts et al. [104]
	USA	*Clostridium novyi*-NT	24 patients	Tumors	-It is possible to infuse *Clostridium novyi*-NT intravenously just once.—An infusion of *Clostridium novyi*-NT increased systemic T-cell responses specific to tumors and produced a transitory systemic cytokine response	Janku et al. [211]
Clinical trials		*Salmonella*	three patients	Solid tumors	showed the cytosine deaminase gene may be transported to malignant tissue via the *Salmonella* bacteria and that the gene can operate (i.e., convert 5-FC to 5-FU) at levels of little more than 3 × 10(7) CFU/m(2)	Nemunaitis et al. [212]
		*Listeria monocytogenes*	90 patients	Pancreatic cancer	Patients with pancreatic cancer had a longer survival rate when heterologous prime/boost combined with cyclophosphamide/GVAX and CRS-207, with very little toxicity	Le et al. [213]
	China	*Clostridium butyricum*	GC patients age 37 to 83	GC	Research indicates that administering *Clostridium butyricum* orally after a gastrectomy can reduce inflammation, boost the immune system, restore microbiota, increase intestinal SCFAs, reduce postoperative complications, and aid patient recovery	Cao et al. [214]

*Abbreviations*: *CRC* Colorectal cancer, *GC* Gastric cancer, *BC* Breast cancer

### Bacteria released substances (toxins or enzymes or bacteriocin) in cancer therapy

**Fig. 2:**
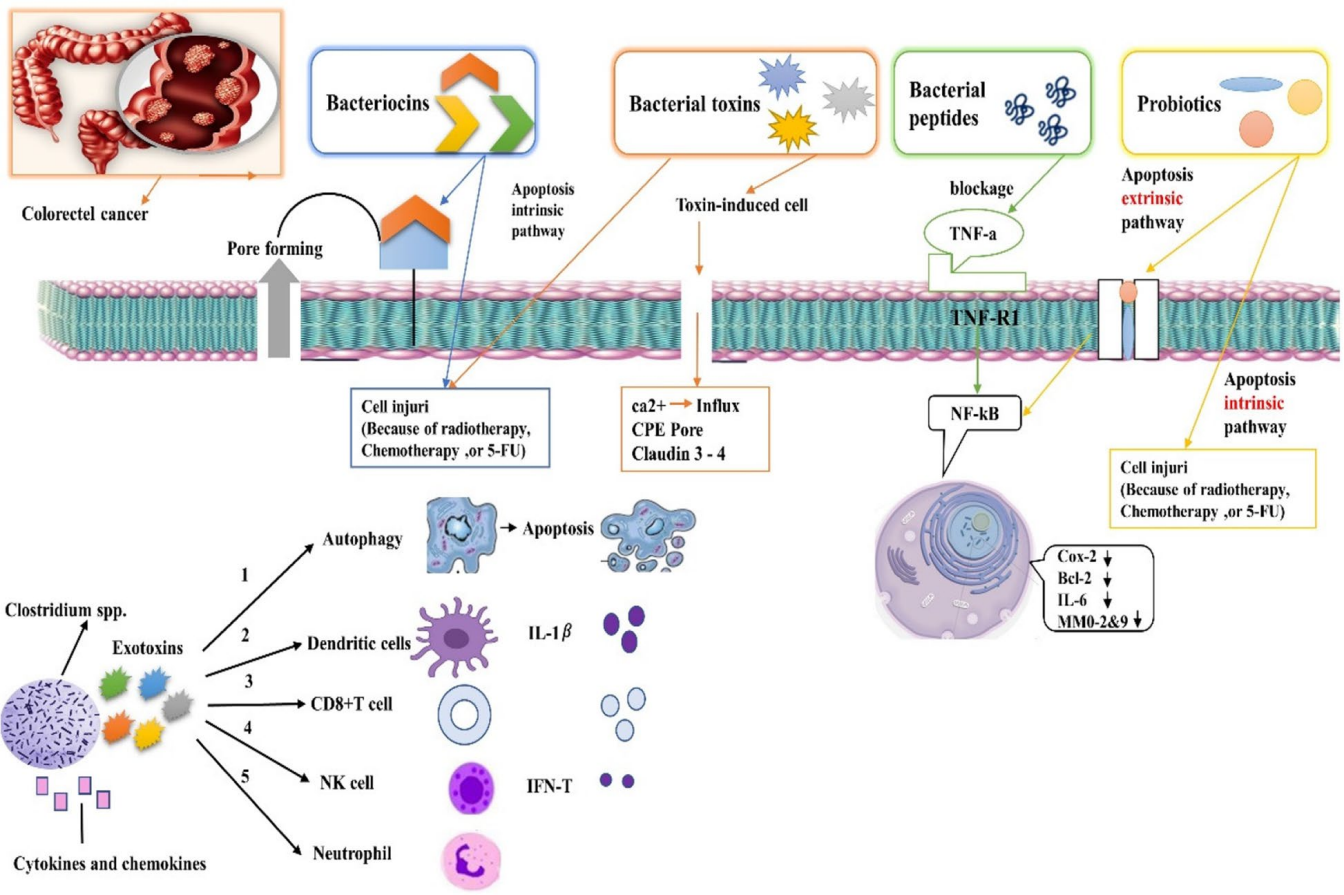
Colorectal cancer: In the bacterial treatment of colorectal cancer, the whole bacterial cell and its metabolites can be used, including probiotics associated with the bacteria, peptides such as bacteriocins, or bacterial toxins. The anti-cancer mechanism of this type of treatment includes: 1) Creating pores in the cell membrane, 2) induction of apoptosis, 3) TNF-α production, 4) inhibition of metastasis. Sometimes molecular sites lead to apoptosis of cancer cells through intrinsic or extrinsic pathways. *C. perfringens* enterotoxin (bacterial toxin) CPE can directly interact with claudin-3 and claudin-4, which are overexpressed in colorectal cancer cell membranes. Another mechanism of the anticancer effect of bacterial toxins is cytotoxicity through the intrinsic pathway of apoptosis. Bacteriocins create membranous adhesions when attached to the membrane and a specific type of cell surface that induces cell lysis and cell death

Numerous compounds generated from bacteria can selectively target cancer cells and provide a cytotoxic impact [215]. Enzymes, peptides, specific secondary bacterial metabolites, and bacterial toxins are examples of cytotoxic agents [216]. In this part, we look at how chemicals secreted by bacteria are used to treat cancer and discuss the significance and uses of these compounds in this area (Fig. 2).

### Bacterial toxins

Bacterial toxins are one of the compounds produced by bacteria and employed in cancer therapy [217]. Certain toxins that are created and released by bacteria have the potential to be cytotoxic or, in less extreme cases, to change apoptosis, differentiation, and proliferation [218]. Through various methods, including cell-cycle arrest and disruption of tumor cell signal pathways, some of them can successfully prevent tumor development [219]. Cytolysin CylA is one of the most well-known bacterial toxins used as an anti-cancer agent [219, 220]. Pore-forming substances called cytolysins cause multimeric holes in cell membranes and aid in the death of cells. *Salmonella typhimurium*, *E. coli*, and *Staphylococcus aureus* are the usual sources of cytolysins [221–224]. Mice given strains of *S. typhimurium* or *E. coli* that produce the CylA toxin showed suppression of tumor development [225, 226]. The diphtheria toxin (DT), a primary virulence factor generated by *Corynebacterium diphtheria*, is another well-known toxin. The toxin exhibits fatal effects on mammalian cells at low doses. Several cancer types have shown improvement in response to therapy with modified DT-based toxins [227, 228]. The two kinds of cytotoxin and enterotoxin found in *Clostridium difficile* toxin can destroy cancer cells by attracting proinflammatory molecules and inducing an immune response [229–231]. Botulinum neurotoxin A, which *Clostridium botulinum* produces, causes apoptosis in BC cell lines T47D and decreases cell growth and proliferation in prostate cancer lines PC-3 and LNCaP [232–235]. The enterotoxin generated by *Clostridium perfringens* possesses anti-cancer properties as well. It binds to the overexpressed claudin-4 receptor on pancreatic cancer cells, causing dose-dependent acute toxicity [236, 237]. Produced by pathogenic *E. coli*, verotoxin 1 (VT-1), also known as Shiga toxin-1 (Stx1), can halt the cell cycle in the colon cancer HCT116 cell line [99, 238]. *Pseudomonas aeruginosa* produces exotoxin A (PE), which inhibits protein synthesis by ADP ribosylation and causes cancer cells to die [239, 240]. Cytotoxic necrotizing factor (CNF), a toxin produced by *E. coli*, stimulates DNA replication and results in the creation of multinucleated cells as a secondary effect of suppressing cell differentiation and inducing death [241, 242].

### Bacterial enzymes

Numerous types of enzymes are produced by bacteria. A few can affect the vital amino acids needed for tumor development [243–245]. By increasing the effectiveness and specificity of treatment, bacterial enzymes have demonstrated promise in cancer therapy [246]. L-asparaginase, an enzyme generated by *Bacillus subtilis*, *Streptomyces*, Erwinia species, or *E. coli*, is one of the bacterial enzymes that is often studied [247]. This enzyme is responsible for catalyzing the hydrolysis of asparagine, which lowers its blood content and kills tumor cells [248]. Treatments for acute lymphoblastic leukemia, neoplasia, lymphosarcoma, and other cancers have demonstrated the efficacy of L-asparaginase [249, 250]. Two more bacterial enzymes that are important for the catabolism of arginine are arginine decarboxylase and arginine deiminase [251, 252]. It was shown that arginine in tumor cells may be consumed by arginine deaminase derived from *Streptococcus pyogenes*, which inhibits the growth of arginine-deficient tumor glioblastoma multiforme [253, 254].

### Bacteriocins

Bacteriocins are primarily recognized as protein molecules produced by certain bacteria that inhibit the development of other bacterial species or maybe eradicate them [255, 256]. Bacteriocins have the occasional ability to stop tumor cell development. Because of its amphiphilic properties and cationic charge, bacteriocin can interact with negatively charged cell membranes to disrupt their integrity and cause cancer cells to undergo apoptosis [257, 258]. As an example, nisin, a well-researched and often utilized bacteriocin generated by *Lactococcus lactis*, has shown antibacterial action against most Gram-negative bacteria as well as being cytotoxic to MCF-7 cells (a cell line used to treat human BC) [259]. In vitro research on colicins, a well-known family of antimicrobial peptides, has also shown anti-cancer potential [184]. Important bacteriocins with antitumor qualities that can be used in cancer therapy include fermenticin HV6b, S2 pyocin, pediocins, nisin A, colicins, and bevacinocin HC5 [260, 261]. These bacteriocins' diverse properties have been examined from various angles and are included in Table 3.

**Table 3:** Bacteriocins in cancer therapy

Name	Origin	Molecular weight (kDa)	Composed of:	Conclusion	References
Bovicin HC5	Streptococcus bovis	2.4	22 amino acids	Only at concentrations higher than the concentration required for its biological activity did bovicin HC5 exhibit cytotoxic effects	[262, 263]
Colicins	*E. coli*	40 to 80 kDa	Colicin Z (151 amino acids)	Modifying the target cell's electric charge distribution (which causes it to die)	[259, 264–266]
Nisin A	*Lactococcus lactis*	3.3	34 amino acids	Through destabilizing cell membranes, inhibiting tumor cell development, pore deformation, changing cell membranes, and increasing ion penetration that tampers with phospholipid organization, it demonstrates its antitumor and anti-metastasis effect	[264, 267–270]
Pediocins	*Bacterium pediococcus acidilactici* MTCC 5101	4.6	44 amino acids	preventing tumor cells from proliferating by upsetting the cycle of cell division	[271–273]
Fermenticin HV6b	*Bacterium Lactobacillus fermentum* HV6b MTCC 10,770	6.7	––––	Its ability to induce vascular endothelial cells to undergo apoptosis, break down DNA, and contract cells exhibits its anti-cancer effect	[264, 274, 275]
S2 Pyocin	*Pseudomonas aeruginosa* 42A	73.9	777 amino acids	It inhibits the production of lipids in cells and interferes with DNA replication to demonstrate its anti-cancer properties	[276–278]

### Biosurfactant

The surface-active components of various structures made by microbes are known as biosurfactants. According to recent reports, biosurfactants can function as anti-cancer agents by blocking some critical signaling pathways, which interfere with the processes that lead to cancer development [279–281]. Furthermore, biosurfactants can activate NK cells, prevent angiogenesis, and cause cancer cells to undergo apoptosis through death receptors [282]. Compared to synthetic analogs, microbial biosurfactants are thought to be less harmful and biodegradable [283]. Among them is *Bacillus safensis* surfactin, which demonstrated antitumoral solid activity against B16F10 murine melanoma cells and T47D BC cells [284]. Another instance is the cyclic lipopeptide viscosin, which was shown to have significant anti-cancer activity and was derived from *Pseudomonas libanensis*. According to the MTT results, viscosin prevented MDA-MB-231 from proliferating in BC cells. Additionally, viscosin stopped the PC-3 M prostate cancer cell line from migrating [285, 286].

### Bacteria can be anti-cancer agents through biofilms

In the extracellular polymeric matrix, bacteria assemble into thick, spatially ordered networks called biofilms that cling to biological and non-biological surfaces. The process of biofilm development is regulated by the quorum sensing phenomenon, which further helps the bacteria to survive in the host cells and evade the host defense immunological system [287, 288]. Furthermore, the development of biofilms may contribute to the advancement of colorectal and colon cancer [289]. Because biofilm can transport treatments and stop the spread of metastatic tumors, it can be used as a possible anti-cancer drug [290, 291]. When *Pseudomonas aeruginosa* was being treated for cancer with hydroxyurea and doxorubicin, anti-cancer drugs stimulated and encouraged the production of biofilms [239, 292]. An inducible DNA damage repair pathway known as the SOS response is activated by the bacteria growing on the cancer cells to evade the drug onslaught [293]. As a result, several distinct bacterial phenotypes emerge that assault or enter the cancer cells. Additionally, the bacteria that coat cancer cells release DNA and various proteins that prevent the tumor from spreading [294, 295]. To create naturally occurring nanowires that may be used as drug carriers for cancer treatment, Kumeria and colleagues [296] synthesized biofilms of the zetaproteobacterium *Mariprofundus ferrooxydans*. When exposed to an alternating magnetic field, biofilm-derived nanowires were discovered to be magnetic nanomaterials that could produce both passive and active trigger responses. Reduced cell viability was a result of the alternating magnetic field-induced hyperthermia. The doxorubicin-loaded biofilm-derived nanowires were examined for their carrier potential and were shown to be cytotoxic to human BC cells (MDA-MB231-TXSA) [296]. T cells play a crucial role in the initiation of biofilm formation, regardless of the underlying causes and the role they play in protecting bacteria from the host's immune response [297]. During cancer treatment, anti-cancer drugs induce biofilm formation, which leads to metastasis formation of bacterial biofilms on cancer cells during the SOS response, leading to the disorder of metastasis [184]. Bacterial biofilm can affect the growth of colon cancer; its progression can regulate cell proliferation by altering cancer metabolism [298]. Macromolecules, such as DNA and proteins, are essential for biofilm formation. These molecules form a protective layer around cancer cells. For instance, adhesion is inhibited by the polysaccharides that *Streptococcus agalactia* releases. Endothelial cells are produced by cancer cells, and this is crucial for the spread of cancer [296, 299]. Protein nanowires derived from bacteria called *Mariprofundus ferroxydans* (As a novel multipurpose medication carrier for the treatment of cancer and hyperthermia, it may be used [296].

### Future perspectives: bacteriobots technique for cancer therapy

**Fig. 3:**
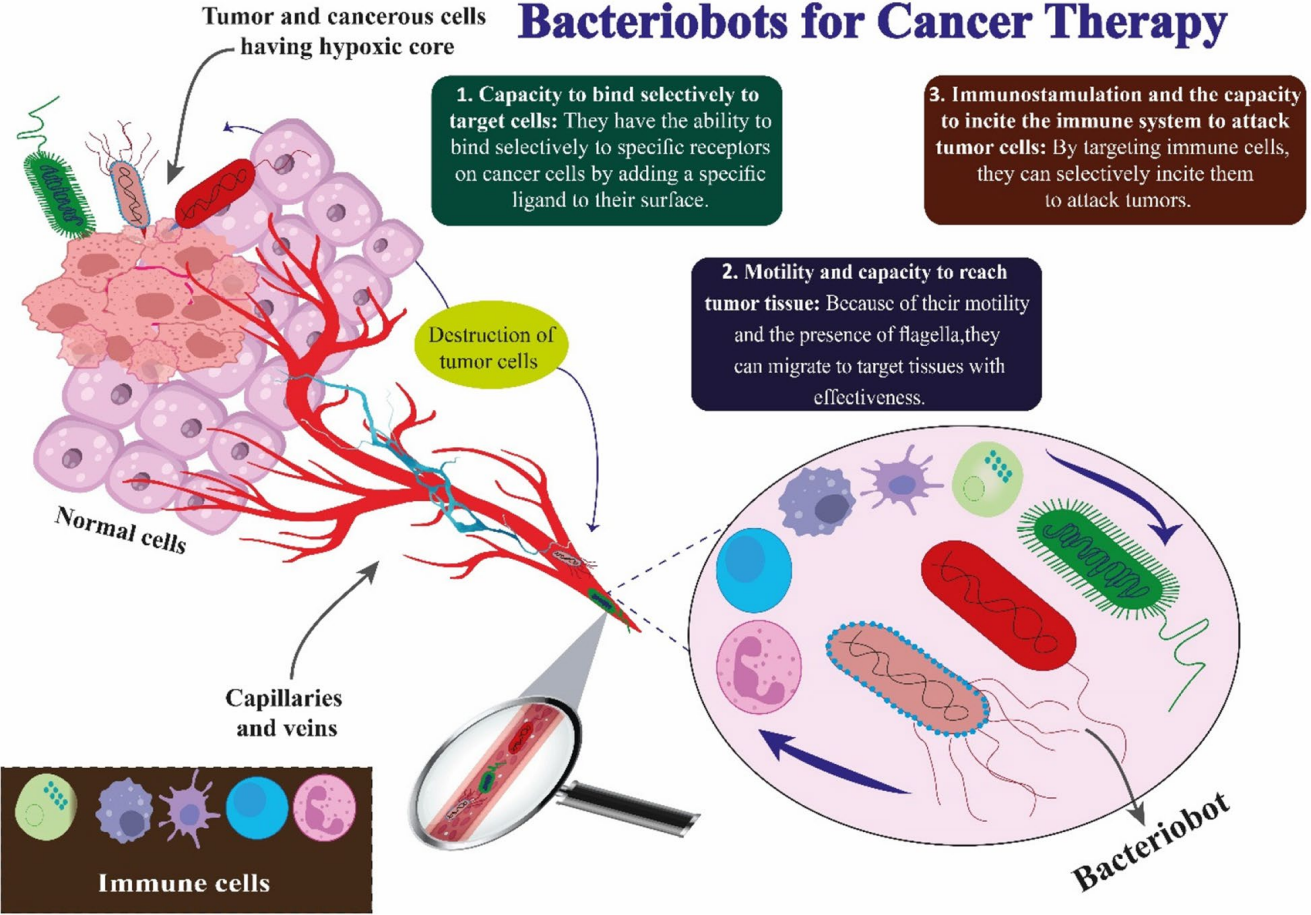
Bacteriobots technique for cancer therapy

In certain publications, "bacteriobot" refers to a novel and inventive theranostic approach utilizing bacteria-based construction for tumor treatment [300, 301]. But, in a broader sense, it may be used for any bacteria that has undergone deliberate modification [302–304]. The effective delivery of drugs to tumor areas is one of the main obstacles in cancer research. Miniature gadgets that actively go towards the tumor, penetrate it, and accumulate there or in surrounding tissues might be an excellent solution to this issue [305, 306]. These devices can be entirely synthetic (chemically and, or physically actuated), comprising only materials, structures, and components that are manufactured by humans, or they can be biologically actuated (cellular microrobots), which are made entirely of human cells and have been carefully designed to have anti-cancer properties (Fig. 3) [307, 308]. Furthermore, hybrid versions that can be pushed by biological or artificial methods (typically biologically actuated) can be built, comprising both artificial and cell-made components [219]. Natural bacteria, especially human commensals, can be used as a vector to deliver a chemotherapeutic chemical directly into the tumor, which might greatly minimize the adverse effects of treatment often associated with conventional chemotherapy [309, 310]. Bacteria can enter the target actively—for example, by using their flagella—or passively—through blood flow. Bacterial flagella can swim up to 300 μm/s and revolve like propellers [311, 312].

Using magnetic fields to guide magnetic particles, irrespective of tumor location, from their application site to the malignancy seems like a promising approach. Bacteria in this situation need to be magnetic field sensitive. One intriguing example is magnetotactic bacteria, which can detect magnetic fields and align their swimming paths along them because they contain naturally occurring magnetic particles called magnetosomes [313–316]. Therefore, a Gram-negative coccus present in the Atlantic Ocean is *Magnetococcus marinus* (MC1). With two bundles of cilia organized at one pole, this microbe can move at a speed of 300 μm/s [317, 318]. This particular bacterium's magnetosomes are chains of magnetite (Fe3O4) particles encased in membranes that originate in the cytoplasm. The bacteria are oriented about the Earth's magnetic field by the presence of magnetite. It might be feasible to target bacteria carrying magnetosomes to the tumor by applying a strong magnetic field similar to that used in MRI (magnetic resonance imaging) procedures. A recent study found that, in comparison to nonguided MC1 bacteria, magnetic guidance significantly increased the tumor formation of MC1-based hybrid microrobots injected intraperitoneally in live mice [318–320]. Recently, it was possible to construct *E. Coli* to sense magnetic fields by forcing the bacterial cells to create iron-rich structures [321]. Enhanced tumor targeting is a noteworthy function that bacterial microrobots may assist with. Cancer cells and their microenvironments take on chemical and physical properties that set them apart from normal cells due to mutations and other genetic and epigenetic abnormalities [322–324].

The bacteria are shielded by the microbeads from opsonization and other potential physiological changes by the body [325]. Since these microbeads come into intimate contact with healthy organs, it is essential to use the right microbeads for creating practical bacterial robots that target tumors [326]. In the end, adding bacterial flagella to microbial granules would ease the agents' delivery to the target region and enable the movement of bacterial robots [327]. *Salmonella typhimurium* was used in one study to create a microbot that was based on bacteria. In this study, bacteria were encapsulated in biocompatible alginate granules and subsequently attached to microbeads as a motility section by *S. typhimurium* flagella. The tests showed that these robotic bacteria could successfully target the tumor [327]. Non-pathogenic *E. Coli* was used as living nanobots to cure cancer in another investigation by Al-Fandi and colleagues [328]. The nano-biosensor devices, spontaneously synthesized and attached to VEGF, were fitted to these living microrobots. The membranes of the tumor cells had an overexpression of the VEGF receptor, which the microbial nanobots were designed to recognize and adhere to [328]. Robotic bacteria are said to migrate to tumors at higher rates than healthy cells, possess better chemotactic motility, and target malignant tissue more effectively. Additionally, the latest breed of bacterial robots serves as micro-sensors and micro-stimuli. This implies they can be used as delivery systems to deliver medications and therapeutic nanoparticles (NPs) to tumors. Bacteriobots might be a novel therapeutic tool for identifying and combating solid tumors [329–331]. The perfect bacterial microrobot should resemble a type of "minicell"—a nanoscale, anucleated, nondividing, metabolically active cell that can translate and transcribe the desired gene. By simply modifying the surface of the minicell with particular antibodies directed against cancer cells that have receptors, minicells ought to encapsulate a broad spectrum of chemotherapeutic and molecular medicines, si/shRNA, antigens, and therapeutic toxins and transport them to cancer cells with precision [332].

### Combination of virotherapy and immunotherapy

A range of viruses could be genetically modified to infect and lyse tumor cells as cloning technology advanced. Viral treatment has succeeded because of our growing understanding of viral mechanisms of action, which include regulating the TME and triggering both innate and adaptive antitumor immunity [333]. Currently being employed as OVs include several viruses, including vesicular stomatitis virus, coxsackievirus, adenovirus, measles virus, reovirus, and HSV [95, 334–336]. OVs elicit both anti-cancer and antiviral immunity. Tumor therapy benefits from antitumor immunity. Based on the idea that the antiviral immune response limits the growth and spread of OVs, host immunological responses have long been thought to be detrimental to the effectiveness of OVs [337–339]. However, it has recently been recognized that the antiviral immune response is advantageous in treating tumors for the first priming of antitumor immunity by OVs [340]. OVs increase innate immunity, turn "cold" tumors into "hot" tumors that impede tumor growth, draw immune cells, and activate systemic anti-cancer adaptive immunity [341, 342]. Reduced IFN activity in conjunction with elevated EGFR and downstream signaling pathways, including PI3K, Ras, and MAPK activation, may allow Ovs to evade the immune system and infect cancer cells while increasing and spreading to create offspring that may ultimately cause tumor cells to die [343–345]. A wide range of viruses thrive in the presence of cancer cells because these cells can evade the body's immune system to live. Because tumor cells do not undergo apoptosis and instead suppress interferon signaling, they have developed into suitable hosts for various viruses. Moreover, laminin, CAR, and CD155 overexpression increases the susceptibility of cancer cells to viral infection. Notably, many of the elements of the TME that give cancer stem cells (CSCs) resilience to conventional therapies are ineffective against OVs [346].

Newcastle disease virus (NDV) infects birds. It is non or less pathogenic to humans and is associated with Type I interferon signaling stimulation, and NDV/ HK84 strain can inhibit hepatocellular carcinoma (HCC) progression in cell lines and also in mice when injected intratumorally [347]. Some strains of reoviruses can replicate in cancer cells selectively and may induce tumor regression in subcutaneous tumor models in animals [348]. Additionally, parvovirus H1 showed partially successful results in metastatic pancreatic cancer in clinical trials [349]. Although it has been attempted to use viral strains that are not known to be associated with any human diseases, due to the potential toxicity and uncontrolled pathogenicity, researchers have turned to genetically modified viruses known as viral vectors [347]. Viral vectors can deliver therapeutic genes, in the case of cancers, tumor suppressors, and oncolytic genes, specifically to the tumor site [350]. Controlled and specified expression of genes and removal of virulent viral genes by genetic engineering techniques have made viral vectors attractive tools for targeting tumors [351, 352]. Several virus families that have been used as the viral vectors include retroviridae, adenoviridae, parvoviridae, herpesviridae, togaviridae, flaviviridae, rhabdoviridae, paramixoviridae, picornaviridae and poxviridae in preclinical and clinical trials [353].Viral vectors have many applications in infectious and non-infectious diseases, prevention, diagnosis, and treatment [354]. For example, one of the next-generation vaccine platforms of SARS-CoV-2 is viral vectors such as Oxford – AstraZeneca (based on chimpanzee adenovirus), Ad5-nCoV (based on adenovirus type 5) and Sputnik V (based on adenovirus types 5 and 26) which are expressed SARS-CoV-2 spike protein as the immunological dominant protein [21]. Human immunodeficiency virus (HIV)/AIDS, a chronic disease that spreads worldwide, is an excellent field of study about viral vectors, especially in preventive and therapeutic vaccines and cure strategies [353]. For example, designing a lentivector containing a mutant form of APOBEC3G and inhibiting infection spread in cell lines or targeting CNS latent reservoirs by a combination of Romidepsin as a latency reversal agent and Adeno-associated virus vector carrying thymidine kinase gene for inducing apoptosis in reactivated cells are just a few examples of the use of viral vectors in the field of HIV/AIDS [355]. As single agents, unarmed or armed OVs have shown excellent safety and potential therapeutic results in tumor therapy [356]. Monotherapies, on the other hand, are unlikely to entirely reverse T-cell function loss induced by tumor heterogeneity and an immunosuppressive microenvironment [334].

In several clinical studies, promising OVs genetically modified with other antitumor agents were able to eradicate tumors [357]. Armed OVs with ICIs plus adoptive T-cell therapy (ACT) have recently demonstrated exceptionally high efficacy by triggering several antitumor steps, such as promoting T-cell expansion and survival, boosting T-cell trafficking to tumors, improving APC function, and reversing T-cell exhaustion [334]. OVs designed to encode ICIs are potential therapeutics. However, the most prevalent approach to treating tumors with ICIs is to utilize ICI antibodies, such as the licensed medications ipilimumab (anti-CTLA-4), pembrolizumab (anti-PD-1), nivolumab (anti-PD-1), cemiplimab (anti-PD-1), avelumab (anti-PD-L1), and atezolizumab (anti-PD-L1) [358]. Despite the effectiveness of these ICIs, it is anticipated that only 12.5% of patients who undergo ICI treatment benefit [359]. The lack or low expression of PD-L1 on tumor cells is one of the most generally recognized explanations for initial resistance to ICI treatment [360]. OVs have been demonstrated to boost PD-L1 levels significantly, which is advantageous to ICI treatment [361]. Tumors become more receptive to ICI treatment when OVs armed with cytokines expand the altered form of the TME into a proinflammatory microenvironment [361]. Pembrolizumab treatment improved the 62% objective response rate with a 33% CR rate in patients with advanced melanoma who received IMLYGIC treatment. Patients also showed increased CD8 + T cells, elevated PD-L1 protein expression, and IFN-γ gene expression in several tumor cell subsets. In patients with advanced, incurable melanoma, similar outcomes were shown in a phase II trial assessing the safety and effectiveness of IMLYGIC with ipilimumab; combinatorial therapy produced a more significant objective response than ipilimumab alone [362, 363]. The findings show that combining ICIs and OVs can increase therapeutic outcomes in cancer patients who have become resistant to ICIs alone [364]. OVs, combined with CAR T-cell and TCR T-cell treatments, T-cell immunotherapies that have been genetically modified have lately demonstrated encouraging clinical results in treating hematologic malignancies [365].

### Clinical trials

Numerous clinical trials have been undertaken to assess the effectiveness and safety of utilizing therapeutic bacteria and viruses in the treatment of cancer. These studies have explored different facets of using microbial agents for anticancer therapies, such as their efficacy in attacking and eradicating cancerous cells, their influence on tumor reduction, and their possible negative consequences on individuals undergoing treatment [366, 367]. A clinical trial in phase 1b investigated the impact of oncolytic virotherapy, Talimogene laherparepvec (T-VEC), in combination with pembrolizumab, an anti-PD-1 antibody, among individuals diagnosed with advanced melanoma. Patients who responded positively to the combined therapy showed a rise in CD8 + T cells, heightened levels of PD-L1 protein expression, and increased IFN-γ gene expression across various cell subsets within tumors following treatment with T-VEC. The response to combination therapy did not correlate with the initial levels of CD8 + T cell infiltration or the initial presence of an IFN-γ signature. Consequently, their research indicated that oncolytic virotherapy has the potential to enhance the effectiveness of anti-PD-1 therapy through modifications to the TME [368]. Prior research has shown that some patients exhibit resistance to PD-1 blockade as a result of a lack of CD8 + T cells in the tumor site [369, 370]. The phase 2 trial involving 692 participants in a similar environment demonstrated a satisfactory safety record; however, it did not achieve the primary progression-free survival (PFS) endpoint of 14.3 months (median; range = 10.3–22.1). In comparison, the PFS for the placebo and pembrolizumab group was 8.5 months (median; range = 5.7–13.5), with a hazard ratio of 0.86 (confidence interval = 0.71–1.04, *p* = 0.13) [371]. Research has demonstrated that JX-594, an engineered oncolytic poxvirus, when infused intravenously, spreads infection across tumors while sparing healthy tissues [372–374]. During phase I/II clinical trials, JX-594 demonstrated favorable tolerability following intravenous administration and did not elicit any dose-limiting toxicities, with the maximum tolerated dose not being achieved [372, 373, 375]. Nevertheless, the administration of JX-594 alongside sorafenib did not demonstrate a significant improvement in survival outcomes during a phase III clinical trial involving individuals with advanced HCC who had not received prior systemic treatment (NCT02562755). T3011 is a modified form of the HSV-1 that has been genetically engineered to encode IL-12 and an antibody targeting PD-1. The latest phase I clinical trial findings indicated that T3011 demonstrated favorable tolerability among patients diagnosed with advanced cutaneous or subcutaneous malignancies (NCT04370587) [376]. In a separate investigation, a new OVV containing a complete monoclonal antibody targeting TIGIT demonstrated enhanced effectiveness in combating tumors and elicited enduring tumor-specific immunological memory [377].

In recent decades, numerous research studies have been undertaken to investigate the utilization of various clostridium species as agents for targeting tumors. One of the most highly regarded options is a genetically engineered strain of *Clostridium novyi-NT*. The toxicity of this strain can be reduced by getting rid of a residential phage that carries the α-toxin. Thus far, phase I and II clinical studies including the attenuated *C. novyi-NT* strain have had favorable results [302, 378]. An example of complete tumor regression following intravenous injection of *C. novyi-NT* spores into a patient with advanced leiomyosarcoma was reported [379, 380]. Clinical trials investigating the potential therapeutic application of *Salmonella enterica* serovar *Typhimurium* (*S. typhi*) for the treatment of melanoma were initiated in 2002 and have progressed to phase I trials as of the present time. Furthermore, the VXM01 antitumor vaccine, derived from the weakened strain of *S. typhi*, has effectively completed phase I clinical trials [381–383]. A clinical study was carried out to assess the efficacy of live *Bifidobacterium tetragenous* bacteria tablets in managing functional constipation in cancer patients undergoing chemotherapy. The study revealed that administering live *Bifidobacterium tetragenous* bacteria tablets proved to be both efficacious and well-tolerated in managing functional constipation in cancer patients undergoing chemotherapy [384]. In a randomized controlled trial conducted in 2021, researchers sought to investigate the impact of synbiotics on bacterial translocation and consequent bacteremia in patients undergoing neoadjuvant chemotherapy for esophageal cancer. The researchers discovered that administering neoadjuvant chemotherapy to patients with esophageal cancer may lead to bacterial translocation and subsequent bacteremia. However, this adverse effect can be mitigated by using synbiotics [385]. In another research, a randomized controlled trial was conducted to assess the impact of inulin, a recognized prebiotic substance, on the advancement and evolution of colon cancer. The findings of the 28-week research study indicated that the consumption of inulin in the diet can effectively inhibit inflammatory processes that contribute to the progression of colon cancer [386].

### Viruses and bacteria: a double-edged sword in cancer

Certain viruses and bacteria, alone or in conjunction with other cofactors, can cause cancer by disrupting critical cellular processes [387]. A growing body of research has demonstrated the tight relationship between the infection of various tumors and microbes such as bacteria and viruses [388, 389]. Every organ's carcinogenesis is also influenced differently by the microbiota, as the host's and the microbes' genotypes impact cancer susceptibility and promotion [390]. HPV, human T-cell leukemia virus type1 (HTLV-1), hepatitis B virus (HBV), hepatitis C virus (HCV), Epstein–Barr virus (EBV), and HHV-8 are among the DNA and RNA viruses that are known to be linked to cancer [77, 391, 392]. We can see that HTLV-1 is the most cancer-causing virus on the list, followed by HPV, HBV, and HCV. HHV-8 and EBV, for the most part, require a co-factor to exhibit their carcinogenic potential, but as global health issues, they have a far more significant impact due to their much higher prevalence. Their viral genomes are smaller (from a few Kb to around 200 Kb) and have less coding power. As a result, they rely on cellular proteins to complete their life cycles and promote viral particle production, affecting various cellular pathways such as DNA repair, proliferation, and apoptosis [393]. Consequently, these viruses induce oncogenesis via a multi-step process that involves tumor-initiating and, or later-stage, tumor-promoting and spreading, as well as apoptosis, the regulation of cell proliferation, and senescence [394, 395]. Although the process of tumor growth is not virus-specific, various viruses can alter different phases of the process [396, 397].

Up until now, only *Helicobacter pylori* (*H. pylori*) has been linked to carcinogenesis through epidemiological data; however, more and more bacteria have been linked to cancer in humans, and research on the human microbiome has revealed a variety of intricate interactions between prokaryotes and their hosts [398, 399]. While the specific molecular mechanisms by which these bacteria affect the cellular pathways that lead to transformation remain largely unknown, mounting data suggests that they may also inhibit p53 functions and impact DNA repair pathways, thereby increasing the accumulation of DNA damage and ultimately promoting cellular transformation as an antitumor progression [400–402]. Studies in animal models that demonstrate a decrease in tumor burden following antibiotic modification of gut microbiota have supported the idea that bacteria play a role in carcinogenesis [403]. According to this theory, in vivo tests using a variety of chemically generated or genetically deficient animal models show that the presence of germs inhibits the development of colonic tumors [404, 405]. More specifically, several patient studies have demonstrated correlations between non-Hodgkin's lymphoma (NHL) in HIV-positive individuals, *Fusobacterium nucleatum (F. nucleatum)* and CRC, *Chlamydia trachomatis* and cervical cancer, and mycoplasmas and prostate and CRC [77, 406]. Furthermore, although the bacterial protein responsible has not yet been identified, infections with several mycoplasmas (*Mycoplasma fermentans*, *arginini*, *hominis*, and *arthritidis*) inhibit p53 activity and collaborate with Ras to cause oncogenic transformation in vitro, firmly establishing them as top bacterial candidates with carcinogenic qualities [407]. Additionally, it has been demonstrated that p53 and p21 expression was decreased in gastric mucosal cells after a prolonged infection with *M. penetrans* in a model of chemically immunosuppressed mice, resulting in pathological alterations [408].

### Limitations and potential drawbacks bacteria and viruses cancer therapy

Bacterial and viral therapies have demonstrated potential efficacy in cancer treatment; however, it is essential to acknowledge the existing limitations and potential disadvantages associated with these treatment modalities. One constraint involves safeguarding unaffected tissues while stimulating immune reactions [366]. Moreover, specific bacteria and viruses can potentially impede the mechanisms responsible for preserving genetic stability and cellular restoration, thereby diminishing the efficacy of therapeutic interventions [409–411]. Another problem encountered in managing cancer through the use of bacteria and viruses is their potential for toxicity [412, 413]. The dosage required to achieve a therapeutic outcome may result in toxicity and adverse effects, while lower dosages may impact the effectiveness of the treatment [412]. The equilibrium between the advantages and safety of the participants in the trial must be upheld. Suitable methodologies and strategies should be implemented to assess the immune response of the individual and the overall therapeutic efficacy [414]. Discrepancies in the tumor architecture between preclinical animal models and human subjects may influence the ability of bacteria to infiltrate and multiply within the tumor [409]. Therefore, it is crucial to optimize both the dosage and method of delivery carefully. Additionally, eliminating bacteria by the immune system before reaching the tumor location can lead to treatment failure [415]. Furthermore, bacterial mutations can potentially produce exacerbated infections and therapeutic loss. Recombinant DNA technology, however, has allayed chiefly the safety worries [412]. Influenza-like symptoms such as fevers and chills have been observed following the administration of OVs, whether locally or systemically, although they are typically mild [362, 416]. These responses are mitigated through the administration of acetaminophen before the commencement of the treatment [83]. Furthermore, specific therapeutic interventions involving the utilization of bacteria and viruses may result in adverse reactions such as elevated body temperature, emesis, and GI disturbances. These adverse reactions have the potential to impact the overall well-being of patients and may lead to the discontinuation or modification of their treatment regimen [184, 417].

## Conclusion

Although bacteria and viruses are the causes of various cancers, they have recently played a significant role in the treatment and reduction of the side effects of cancer drugs. Many studies are conducted on the treatment of cancer using bacteria and viruses. Chemotherapy involves a lot of costs for patients. Therefore, using the derived compounds of microorganisms and viruses has attracted the attention of many people. Among the therapeutic methods of OVs, there has been significant progress in cancer treatment. Various are used in this field. Viral vectors show their effect in the treatment of malignancy that these vectors are immune modulators, influential factors on tumor suppressor genes and oncogenes Clinical trials of some of these treatment methods have been done. The anti-cancer potential of bacteria and viruses increases due to the anti-oncogene or immune antigen properties and the use of modified antitumor agents in combination with therapeutic processes. The role of bacteria in cancer treatment has grown a lot in the last few years, and the substances coming out of bacteria and secretions such as toxins, enzymes, bacteriocins, and biosurfactants have played a significant role in the treatment of cancer. The effective delivery of drugs to tumor areas is one of the main obstacles in cancer research. Miniature tools called "bacteriobot" that actively go to the tumor, penetrate it, and accumulate there or in the surrounding tissues, maybe a good solution to this problem. This type of treatment is promising, cost-effective, and without side effects. Promotion and Its development need more and more extensive studies.

## Data Availability

No datasets were generated or analysed during the current study.
